# Association between expression of the Bone morphogenetic proteins 2 and 7 in the repair of circumscribed cartilage lesions with clinical outcome

**DOI:** 10.1186/1471-2474-11-170

**Published:** 2010-07-29

**Authors:** Hagen Schmal, Philipp Niemeyer, Jörn Zwingmann, Fabian Stoffel, Norbert P Südkamp, Alexander T Mehlhorn

**Affiliations:** 1 Department of Orthopedic Surgery, University of Freiburg Medical Center, Hugstetter Str. 55, D-79106 Freiburg, Germany

## Abstract

**Background:**

Although there is much known about the role of BMPs in cartilage metabolism reliable data about the *in vivo *regulation in natural and surgically induced cartilage repair are still missing.

**Methods:**

Lavage fluids of knee joints of 47 patients were collected during surgical therapy. 5 patients had no cartilage lesion and served as a control group, the other 42 patients with circumscribed cartilage defects were treated by microfracturing (19) or by an Autologous Chondrocyte Implantation (23). The concentrations of BMP-2 and BMP-7 were determined by ELISA. The clinical status was evaluated using the IKDC Score prior to and 1 year following the operation.

**Results:**

High level expression in the control group was found for BMP-2, concentrations of BMP-7 remained below detection levels. No statistical differences could be detected in concentrations of BMP-2 or BMP-7 in the lavage fluids of knees with cartilage lesions compared to the control group. Levels of BMP-7 did not change after surgical cartilage repair, whereas concentrations of BMP-2 statistically significant increased after the intervention (p < 0.001). The clinical outcome following cartilage regenerating surgery increased after 1 year by 29% (p < 0.001). The difference of the IKDC score after 1 year and prior to the operation was used to quantify the degree of improvement following surgery. This difference statistically significant correlated with initial BMP-2 (R = 0.554, p < 0.001) but not BMP-7 (R = 0.031, n.s.) levels in the knee joints.

**Conclusions:**

BMP-2 seems to play an important role in surgically induced cartilage repair; synovial expression correlates with the clinical outcome.

## Background

Circumscribed cartilage defects are considered as an initial event in the progress of osteoarthritis (OA) [1]. In the last decades different methods have been developed for treatment of this pathology. The Autologous Chondrocyte Implantation (ACI) and microfracturing are regarded as established procedures with documented success in prevention of OA-development. Despite clinical improvement one-third of the patients show early radiographic signs of OA five years after surgery independent from used kind of surgical management [2]; this indicates a certain potential for further necessary treatment perfection. Even though there is much known about cartilage metabolism including significant regulating mediators reliable data about *in vivo *regulation of natural cartilage repair and consequences of surgical interventions are still missing. Measurement of synovial mediator levels in the course of cartilage surgery seems to be a sufficient way to verify the so far collected data of *in vitro *or animal experiments. Therefore, this clinical study was initiated, in which lavage fluids of knee joints with cartilage lesions were prospectively collected and cytokine content was analyzed. After publication of the results gained for the regulators of cartilage metabolism bFGF and IGF-I [3], this article focuses on the role of the Bone morphogenetic proteins 2 and 7 (BMP-2, BMP-7) that both are recognized as candidate growth factors with good potential in cartilage tissue engineering as well as cartilage repair.

BMP-2 and BMP-7 belong to the transforming growth factor-beta (TGF-β) superfamily, consisting of TGF-βs, growth differentiation factors, BMPs, activins, inhibins, and glial cell line-derived neurotrophic factor [4]. BMPs have been identified as very potent inducers of bone, but since then it has become evident that their function is not limited to skeletal development [5]. BMP-2 expression is not only found in mesenchymal condensation in embryonic development [6], but is also able to induce chondrogenesis in human mesenchymal stem cells in culture [7]. For cartilage reparative reasons, BMP-2 can be used to induce chondrogenesis by coating scaffolds with BMP-2 before implantation [8]. Thereby, the scaffold itself can be replaced by the original tissue. This can be combined with culturing mesenchymal stem cells or tissue specific cells on the coated scaffold to gain *de novo *tissue formation in the scaffold [9]. Mechanical injury was found to upregulate BMP-2 as well as BMP-2 signaling in human cartilage explants [10]. This could indicate that BMP-2 is upregulated as a reparative response but could also indicate that BMP-2 is merely upregulated as a pathological side effect, thereby further stimulating injury. BMP-7, also known as osteogenic protein-1 (OP-1) has demonstrated a great potential in bone repair applications. Both BMPs received the regulatory approval as commercially available proteins supporting bone repair i.e. in case of delayed union. It has been shown that BMP-7 also exhibits characteristics as a cartilage anabolic factor because of the ability to induce matrix synthesis and promote repair in cartilage. Data collected so far suggest a significant role for BMP-7 in cartilage repair concerning both articular and disc cartilage applications [11].

The purpose of this study was the *in vivo *evaluation of the potentially chondro-protective and chondro-anabolic cytokines BMP-2 and BMP-7 in knees with circumscribed cartilage lesions and to determine if the cytokine profiles correlate with the clinically assessed knee function. Since the expression patterns for aggrecan, bFGF, IGF-I, and IL-1β and the regulation of the intraarticular total protein content have already been characterized and published [3], correlations of these proteins with the clinical outcome were evaluated in addition to the analysis of BMP-2 and 7. Furthermore, the question should be answered whether surgical procedures of cartilage regeneration lead to an up-regulation of both BMPs that in future might be used as a prognostic factor or to support cartilage healing.

## Methods

### Study design

The study was performed as previously described [3]. Briefly, 47 patients were enrolled in a prospective clinical trial between August 2006 and September 2007. Selection of patients followed the criteria as defined beneath.

Inclusion criteria: performance of an arthroscopy of the knee joint, patients in the control group had no cartilage lesion in MRI and diagnostic arthroscopy, patients undergoing microfracturing or ACI had full thickness cartilage lesions graded III and IV according to ICRS classification [12] of various size, agreement to participate in the study, age > 17 years and < 66 years (as recommended [13])

Exclusion criteria: alcohol or drug abuse, mental retardation with incapability to complete the necessary self-reports, joint effusion > 30 ml, persistent knee instability, infection

The study was approved by the Ethical board of the University of Freiburg (AN-EK-FRBRG-335, study number DRKS00000365 and UKF001822). An informed consent was obtained from every subject included in the study.

### Operation protocols

The ACI surgical technique has been well defined in numerous publications [13-15]. In all patients a matrix (Chondro-Gide^®^, Geistlich Biomaterials, Wolhusen, Switzerland) associated technique for chondrocyte fixation has been used. Microfractures were generated with specially bent awls (Chondropic™, Arthrex^®^, Karlsfeld, Germany) by creating V-shaped perforation holes with a diameter of 1.5-2 mm at a distance of 3 mm (3-4 holes/cm^2^) [16]. The applied kind of cartilage surgery was chosen depending on defect size and depth according to the schema of therapy as previously published [3].

### Specimen collection

Synovial lavage fluids of knee joints of patients undergoing surgery were intraoperatively collected. Before starting the procedure, 20 ml of sterile physiologic saline was instilled into the joint cavity. The saline was mixed within the joint by repeated passive flexion-extension and repeated manipulation of the supra-and infrapatellar regions, and then was aspirated as described by Geborek et al.[17]. This method has been successfully used by a variety of other groups [18-20]. The total volume aspirated was recorded (6.0-10.5 ml). Specimen were centrifuged in order to separate the cells and then stored frozen at -80°C until analyzed. An intraarticular drainage was usually placed; the collected fluid in the drainage bottles was used for analysis of mediator concentrations at day 1 and 2 post surgery. Drainages were removed according to medical necessity defined by the secretion volume per day (< 50 ml/24 h).

### Characterization of patients

5 patients undergoing a diagnostic arthroscopy for unspecific knee complains had no cartilage lesion and served as a control group, in case of the other 42 patients the cartilage defects were treated by microfracturing (19 patients) or by an Autologous Chondrocyte Implantation (23 patients). No patient was operated for a fracture. The average age of the patients with cartilage lesions was 42 ± 10 years, the gender distribution was equal (21/21). The average age of the control group was 30 ± 12 years; the male individuals slightly prevailed (2/3). The body mass index (BMI) of the intervention group was 26.9 ± 3.5, the BMI of the control group was 25.0 ± 3.74. Outcome measures were the Lysholm Score [21], the IKDC Score [22], the Noyes Score [23], the Medical Outcomes Study Short Form-36 (SF-36), and visual analog scales (VAS) for knee pain strength and frequency. Of the 42 patients with surgically treated cartilage defects initially entered into the study protocol, 1-year follow-up data were available in 38 patients (84%). Four patients, two in each intervention group, refused the postoperative follow-up. The questionnaire was done not earlier than after 12 months and not later than 13 months after surgery.

### Grading of cartilage lesion

The amount of chondral damage was graded from 0 to 4 based on the ICRS classification [12] Grade 0 represents normal articular cartilage and grade I shows superficial lesions as soft indentation and/or superficial fissures and cracks. A grade II defect is a partial-thickness defect; it features lesions extending down to less than 50% of cartilage depth. With grade III defects, there are cartilage defects extending down to more than 50% of cartilage depth as well as down to the calcified layer, and down to but not through the subchondral bone. Blisters are included in this grade. In grade IV injuries, the subchondral bone is exposed and ruptured. The total area of chondral defect per patient was calculated by adding the regions with grad III and grade IV lesions. According to this standardized choice of treatment the average defect sizes were 3.4 ± 2.0 cm^2 ^in case of microfracturing, and 6.1 ± 2.6 cm^2 ^in case of ACI (p < 0.001).

### ELISAs for BMP-2, BMP-7, bFGF, IGF-1, IL-1β, Aggrecan, BCA (bicinchoninic acid) Protein Assay

In order to measure concentrations of the indicated proteins, commercially available ELISA kits provided by R&D Systems (Wiesbaden-Nordenstadt, Germany) for BMP-2, BMP-7, bFGF, IGF-I, IL-1β and BioSource (BioSource Deutschland GmbH, Solingen, Germany) for aggrecan were used according to the manufacturers' instructions. Briefly, the assay employs the quantitative sandwich enzyme immunoassay technique. A specific MAb was pre-coated onto a microplate. Supernatants were applied to the wells and, after washing, an HRP-conjugated specific Ab was added to the wells. Following the next wash, color development was proportional to protein concentration and was calculated by comparison with a standard. A colorimetric method was used in order to quantify total protein amount in the lavage fluids. The bicinchoninic acid (BCA) assay was available in kit form from Pierce (Rockford, Ill., USA) and was used according to the manufacturers' instructions. In principle, BCA serves the purpose of the Folin reagent in the Lowry assay, namely to react with complexes between copper ions and peptide bonds to produce a purple end product. Extinction was read at 562 nm within one hour.

### Statistics

All values were expressed as mean ± standard deviation. Data sets were examined with one-and two-way analysis of variance (ANOVA) and individual group means of protein or cytokine concentrations were then compared with the unpaired or paired Student's t-test, individual group means of scores were compared with the Wilcoxon rank sum test. Normal probability plots were done on all data sets and correlation determined by either calculating the Pearson (R)-or the Spearmen (Rho) coefficient depending on distribution. The power for comparing two means based on the normal approximation method was reported where indicated. Statistical significance was defined when P < 0.05.

## Results

### Study parameters

The clinical outcome following regenerative cartilage surgery after 1 year was evaluated using the IKDC and the Lysholm Score. The IKDC Score increased by 29% for all patients undergoing either an ACI or microfracturing from 34.6 ± 15.1 points to 49.1 ± 17.5 points (n = 38, p = 0.0002). The increase for patients treated by ACI was 29% (n = 21, p = 0.0029), for patients undergoing microfracturing 30% (n = 17, p = 0.026). The Lysholm Score increased by 21% for all patients undergoing either an ACI or microfracturing from 52.9 ± 21.1 points to 67.4 ± 19.1 points (n = 38, p = 0.004). The increase for all ACI patients was 18% (n = 21, p = 0.03), for all patients treated by microfracturing 26% (n = 17, p = 0.03). Furthermore, sports activities were evaluated using the Noyes Score; a trend for an increase without statistical significance (preoperative 215 ± 74.81 points to postoperative 235 ± 58.99 points, n = 38, p = 0.06) was seen when assessing all patients together independent on kind of surgery. The effect of operative treatment on pain perception was examined using a visual analogue scale. Both pain-strength and pain-frequency improved statistically significant by 30% (6.5 ± 2.0 vs. 4.5 ± 2.4, n = 38, p = 0.00025) and 25% (7.6 ± 2.1 vs. 5.7 ± 2.2, n = 38, p = 0.00029), respectively. In order to evaluate the influence of the knee function improvement on life quality the SF36 was used. An increase for the physical health (34 ± 9 vs. 38 ± 10, n = 38, p = 0.08, 9.3%) and the mental health (50 ± 11 vs. 51 ± 11, n = 38, p = 0.68, 1.4%) was found without statistical significance. The data are summarized in table 1.

**Table 1:** Overview about clinical outcome parameters

Score	Preoperatively	Postoperatively	P	Difference
**IKDC**	34.6 ± 15.1	49.1 ± 17.5	**<0.001**	29%

**Lysholm**	52.9 ± 21.1	67.4 ± 19.1	**<0.01**	21%

**Noyes**	215 ± 75	235 ± 59	0.06, n.s.	8.5%

**Pain Strength (VAS)**	6.5 ± 2.0	4.5 ± 2.4	**<0.001**	30%

**Pain Frequency (VAS)**	7.6 ± 2.1	5.7 ± 2.2	**<0.001**	25%

**SF36 pcs (physical health)**	34 ± 9	38 ± 10	0.08, n.s.	9.3%

**SF36 mcs (mental health)**	50 ± 11	51 ± 11	0.68, n.s.	1.4%

Overview about clinical outcome parameters comparing preoperative situation with the status after 1 year (VAS evaluation based on a visual analog scale, physical component summary -pcs and mental component summary mcs)

### BMP-2

**Figure 1:**
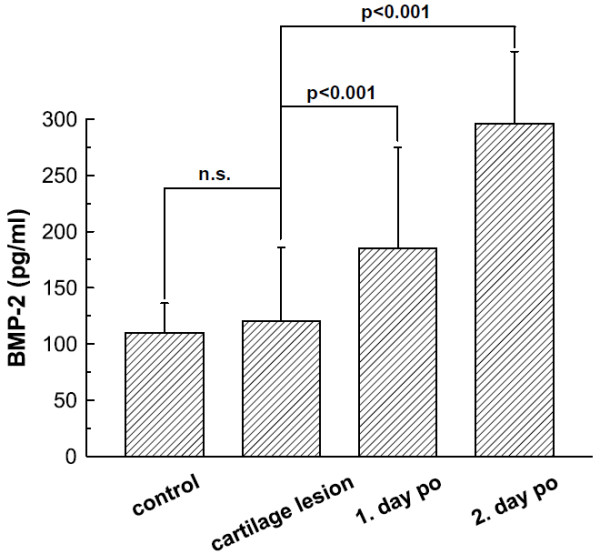
**BMP-2 Course**. BMP-2 content in lavage fluids of knee joints without cartilage lesions (control, n = 5), with cartilage damage (n = 42), following cartilage surgery at day 1 (n = 28) post operation (po), and following cartilage surgery at day 2 (n = 6) post operation. Groups were compared as indicated by the connecting lines, significance levels are specified.

The average concentration of BMP-2 in the lavage fluids of knee joints with cartilage lesions was 120.60 ± 65.22 pg/ml (n = 42), this was not statically significant different from the concentrations in the knees with intact cartilage (110.07 ± 26.16 pg/ml, n = 5, p = 0.72, power 9.43%). BMP-2 expression did not correlate with cartilage defect size; average expression in both intervention groups (ACI and microfracturing) did not statistically significant differ (121.90 ± 83.44 pg/ml vs. 119.03 ± 34.30 pg/ml, p = 0.14). At day 1 following surgery an average BMP-2 concentration of 185.07 ± 89.98 pg/ml (n = 28) was measured what means a statistically significant increase (p < 0.0001, power 91.3%) of BMP-2 levels after surgery. BMP-2 concentrations in the knee joints remained at a high level compared to baseline at day 2 following surgery (296.14 ± 64.19 pg/ml, n = 6, p < 0.0001, power 100%, figure 1).

### BMP-7

**Figure 2:**
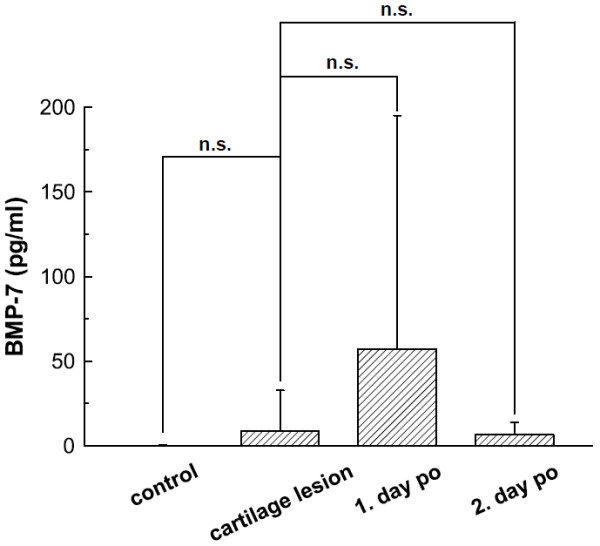
**BMP-7 Course**. BMP-7 content in lavage fluids of knee joints without cartilage lesions (control, n = 5), with cartilage damage (n = 42), following cartilage surgery at day 1 (n = 28) post operation (po), and following cartilage surgery at day 2 (n = 6) post operation. Groups were compared as indicated by the connecting lines, significance levels are specified.

In all knees of the control group concentrations of BMP-7 did not reach detection levels. In the knees with chondral defects the average BMP-7 concentrations were very low (8.56 ± 24.22 pg/ml), in 26 knees with cartilage defects BMP-7 concentrations were below the detection level (figure 2). BMP-7 expression did not correlate with cartilage defect size; average expression in both intervention groups did not statistically significant differ (0.01 ± 0.22 pg/ml vs. 8.56 ± 24.22 pg/ml, p = 0.44, power 57.94%). There was no statistically significant change in BMP-7 levels at day 1 post surgery (8.56 ± 24.22 pg/ml vs. 57.25 ± 137.89 pg/ml, n = 28, p = 0.09, power 46.56%), and no significant difference of BMP-7 levels comparing day 1 and day 2 (6.53 ± 7.23 pg/ml, n = 6, p = 0.26, power 5.98%) after the operation.

### Correlation of cytokine levels with clinical parameters

**Figure 3:**
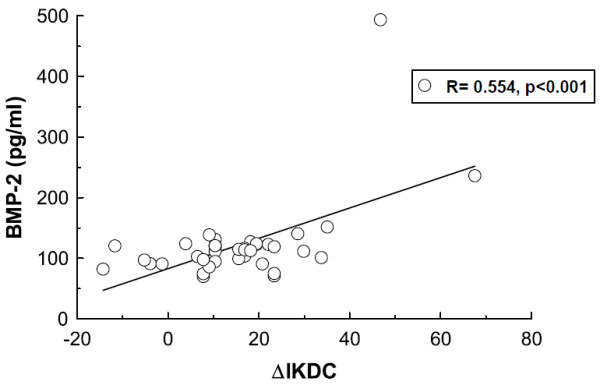
**Association BMP-2 and IKDC Score**. Significant positive correlation between differences of the IKDC Score evaluated initially and after 1 year and initial intraarticular BMP-2 concentrations in all patients undergoing ACI or microfracturing (R = 0.554, p < 0.001)

For the analysis of a possible association between initial synovial cytokine levels and the clinical outcome after 1 year the statistical correlation was calculated. The clinical outcome was defined as the difference of the IKDC or the Lysholm Score after 1 year and prior to the operation that mirrors the individual improvement for each case. There was a statistically significant medium correlation between initial BMP-2 levels and the IKDC Score differences with a Pearson coefficient of 0.554 (p = 0.0003, figure 3), and a significant low correlation of BMP-2 concentrations with the Lysholm Score differences with a Pearson coefficient of 0.378 (p = 0.019). The analysis of the treatment subgroups showed a high correlation of BMP-2 levels with the IKDC Score differences in the patients treated by microfracturing (R = 0.822, p = 0.00005) and a medium correlation for the patients treated by ACI (R = 0.535, p = 0.01). Furthermore, the correlation analysis has been performed for the total protein content and the concentrations of aggrecan, bFGF, IGF-I, and IL-1β (n = 38). None of these intraarticular measured proteins demonstrated a statistically significant association with the clinical outcome defined by the differences of the IKDC Scores or the Lysholm Score (table 2). Quantification of these cytokines and data about postoperative regulations have already been published [3]. Neither synovial BMP-2 nor BMP-7 levels correlated with age (n = 47, R = 0.26, p = 0.29 and R = 0.06, p = 0.60) or BMI (n = 47, R = 0.10, p = 0.50 and R = 0.22, p = 0.13).

**Table 2:** Overview about the correlation strength between the changes of the IKDC Score and the initial intraarticular cytokine levels

Cytokine	R/*Rho*	P
**Protein**	-0.1174	n.s.

**Aggrecan**	-0.1256	n.s.

**BMP-2**	**0.5537**	**<0.001**

**BMP-7**	*0.0307*	n.s.

**IL-1β**	*0.0567*	n.s.

**bFGF**	-0.2218	n.s.

**IGF-I**	*-0.1013*	n.s.

Overview about the correlation strength between the changes of the IKDC Score evaluated initially and after 1 year with initial intraarticular cytokine levels of BMP-2, BMP-7, IL-1β, bFGF, IGF-I, aggrecan and the total protein content. The only statistically significant correlation was found in case of BMP-2 (R-Pearson coefficient, *Rho Spearmen coefficient*).

### Correlation of BMP levels with other cytokines

In order to look for possible regulative associations between the investigated cytokines the statistical correlation between intraarticular levels of IL-1β, IGF-I, bFGF and BMP-2 and -7 was calculated. There was no statistically significant correlation between synovial concentrations of IL-1β, IGF-I, bFGF and the examined BMPs (n = 47, p > 0.08 for all correlations).

## Discussion

Many *in vitro *studies and animal experiments gave significant insights into the role of BMP-2 and BMP-7 in cartilage metabolism and repair; however, data about *in vivo *regulation in humans are ambiguous or still missing for certain clinical situations. Therefore, data about intraarticular levels of BMP-2 and BMP-7 in knees with circumscribed cartilage lesions and their correlation with clinical scores are introduced. Although for both examined BMPs anabolic effects on cartilage were described the data presented suggest a more heterogeneous picture.

Our data demonstrate significant levels of BMP-2 in the synovial fluid of all knees without dependency of the presence or the size of a cartilage lesion. This generally indicates a role for BMP-2 in joint metabolism. Furthermore, increased concentrations of BMP-2 were measured following the cartilage regenerating operation. This might be explained as a consequence of the surgical manipulation of the cartilage defect boarder and the arthrotomy as it has been shown for bFGF, IGF-I or IL-1β [3]. But BMP-2 was the only intraarticular cytokine which correlated with the degree of clinical improvement measured by the IKDC Score. Since it has been shown that the clinical outcome correlates with the degree of cartilage regeneration [24] it may be concluded that BMP-2 plays a significant role in cartilage repair and metabolism. This is in concordance with other studies showing BMP-2 stimulated murine proteoglycan synthesis and BMP-2 induced enhancement of collagen type II expression in chondrocytes seeded in alginate [25,26]. Also, in species like rats and humans, BMP-2 was able to stimulate the chondrogenic phenotype on the mRNA level and induced cartilage extracellular matrix proteoglycan production [27,28]. Further studies [29] have added a partial catabolic effect on cartilage ECM indicating a regulative role for BMP-2 in ECM maintenance, especially during inflammatory induced turnover. This was confirmed by a study that demonstrated a decisive role for BMPs in osteophyte formation and synovial thickening during OA [30], although the presented data do not allow concluding that BMP-2 plays an important role in the onset of OA, because healthy patients also expressed intraarticular BMP-2. Similar to the effects of BMP-2 stimulation of cartilage-specific extracellular proteins as collagens type II and VI, aggrecan, decorin, fibronectin, hyaluronan [31-34] has been shown *in vitro *for BMP-7 (OP-1). It induced anabolic responses in normal and OA chondrocytes from both young and old donors and did not cause expression of cartilage hypertrophy markers or changes in their chondrogenic phenotype [35-37]. BMP-7 was synthesised by human articular chondrocytes [36,38] of different age and various degrees of degeneration including OA, but the level of gene and protein expression was dramatically reduced with cartilage aging and degenerative destruction [36,37]. BMP-7 was mainly localized in the cartilage layer [38], but was also detected in the synovial knee fluid of patients with OA and rheumatoid arthritis (RA) [18]. This articular distribution suggests a decisive paracrine effect of BMP-7, correlating with the finding that intraarticular measured levels are lower than the effective concentrations determined *in vitro *[39]. In conformity with our data the mature and active BMP-7 could not be found in human synovial fluid of normal knees in a recently published study [18]. The degree of degenerative changes seems to influence the intraarticular concentration and possibly induces the conversion of a BMP-7 pro-form into the mature and active protein [18]. This is supported by the data of Honsawek et al., who described increased BMP-7 levels in knees of patients with progressive OA [19] The circumstance that in our trial only patients with circumscribed cartilage lesions were included (and patients with advanced OA were excluded) may make clear why synovial concentrations were mainly found below the detection level and, therefore, did not correlate with the clinical score. Since for both examined BMPs pro-forms [40] have been described, the role and function of these precursor proteins still need to be analysed.

The statistically significant correlation of BMP-2 levels with the clinical outcome was only found using knee specific scores. In contrast, scores reflecting overall live quality (SF 36) or physical activity (Noyes) failed to show this association. This might be explained by the fact that these scores are influenced by much more parameters than knee function. That the aspect knee functionality plays an important role is shown by the difference between the physical and the mental component summary of the SF 36. Inclusion of more patients could possibly lead to a statistical significance regarding live quality or overall physical activity.

Although cartilage repair and inflammatory joint reactions are usually associated with pain and loss of function [41], this study could neither demonstrate regulatory associations between both examined BMPs and the inflammation-triggered IL-1β nor between BMPs and the mediators of cartilage metabolism IGF-I and bFGF. This might be explained by the fact that direct regulations *in vivo *are rare; usually cytokines are controlled on different regulatory levels making a distinguished mediator release dependent on several affecting components possible.

The study does not allow drawing any conclusions how BMP-2 is acting; we even may not determine what part of the operation -arthrotomy or cartilage surgery itself-induces the increase of BMP-2 levels. It also remains unclear what molecular mechanisms are behind the observed association. But the spectrum of BMP-2 effects concerning cartilage repair and differentiation is rapidly growing [42,43]. We also may speculate that BMP-2 plays a role in nervous tissue regeneration, thereby influencing pain perception. A further interesting aspect is the question, whether BMP-2 concentrations are linked to differences in bone turnover induced by damage of the subchondral bone layer during surgery. This hypothesis would be supported by the better correlations seen in patients undergoing microfracturing compared to ACI patients. After all, the study does not allow explaining the BMP-2 induced reactions. This limitation is caused by the plain observational characteristic of the study. We speculate that the cartilage in the near surrounding of the defect and the synovia may play an important role controlling BMP-2 release and that the assembly of the different receptors influences BMP-driven effects [44]. Therefore, a histology study was initiated in order to localize BMPs and their receptors in the different regions of interest in the joint. This will give the possibility to include other promising representatives of the BMP-family with cartilage influencing properties as BMP-4 [45].

## Conclusions

Strong evidence has been collected so far that both examined BMPs play a significant role in cartilage repair. Our data could confirm intraarticular presence of BMP-2 in patients with circumscribed cartilage lesions. The concentrations were increased following cartilage regenerating surgery and statistically significant correlated with improvement of functional knee scores. Therefore, BMP-2 seems to play a significant role in cartilage maintenance and repair.

## List of Abbreviations

ACI: Autologous Chondrocyte Implantation; ANOVA: analysis of variance; BCA: bicinchoninic acid; bFGF: basic Fibroblast growth factor; BMI: Body Mass Index; BMP: Bone morphogenetic proteins; ECM: Extracellular matrix; ICRS: International Cartilage Repair Society; IGF-I: Insulin-like growth factor-I; IKDC: International Knee Documentation Committee; IL-1β: Interleukin-1β mcs mental component summary; OA: osteoarthritis; OP-1: Osteogenic protein-1; PCS: physical component summary; PO: post operation; SF-36: Medical Outcomes Study Short Form-36; TGF-β: Transforming growth factor-β; VAS: Visual analog scales.

## Competing interests

All authors disclose any financial and personal relationships with other people or organizations that could potentially and inappropriately influence (bias) their work and conclusions.

## Authors' contributions

HS was responsible for the conception and the final design of the study, for obtaining of funding, the analysis and interpretation of the data, tutorial of co-authors, and writing of the article. PN performed most operations und by this provided study material, acquired patients, and contributed to data management with his statistical expertise. JZ contributed to the analysis and interpretation of the data, and supported the laboratory part of the study. FS was responsible for the collection, assembly and management of data, performed the ELISAs, and calculated the scores and the descriptive statistics. NPS was involved in the conception and design of the study and the trial protocol, provided study material, contributed to obtainment of funding, and gave administrative support. AM was involved in the conception and the design of the study and the trial protocol and gave significant input in the fundamental considerations of the role of BMP-2 (and its pro-form), and also contributed to the obtainment of funding. All authors read and approved the final manuscript.

## Pre-publication history

The pre-publication history for this paper can be accessed here:

http://www.biomedcentral.com/1471-2474/11/170/prepub

## References

[B1] Cicuttini F Ding C Wluka A Davis S Ebeling PR Jones G Association of cartilage defects with loss of knee cartilage in healthy, middle-age adults: a prospective study Arthritis Rheum 2005 52 2033 2039 10.1002/art.21148 15986359

[B2] Knutsen G Drogset JO Engebretsen L Grontvedt T Isaksen V Ludvigsen TC Roberts S Solheim E Strand T Johansen O A randomized trial comparing autologous chondrocyte implantation with microfracture. Findings at five years J Bone Joint Surg Am 2007 89 2105 2112 10.2106/JBJS.G.00003 17908884

[B3] Schmal H Mehlhorn A Stoffel F Kostler W Sudkamp NP Niemeyer P In vivo quantification of intraarticular cytokines in knees during natural and surgically induced cartilage repair Cytotherapy 2009 11 1065 1075 19877994 10.3109/14653240903219130

[B4] Minas T Nehrer S Current concepts in the treatment of articular cartilage defects Orthopedics 1997 20 525 538 9195635 10.3928/0147-7447-19970601-08

[B5] Wolfman NM Hattersley G Cox K Celeste AJ Nelson R Yamaji N Dube JL DiBlasio-Smith E Nove J Song JJ Ectopic induction of tendon and ligament in rats by growth and differentiation factors 5, 6, and 7, members of the TGF-beta gene family J Clin Invest 1997 100 321 330 10.1172/JCI119537 9218508 PMC508194

[B6] Ducy P Karsenty G The family of bone morphogenetic proteins Kidney Int 2000 57 2207 2214 10.1046/j.1523-1755.2000.00081.x 10844590

[B7] Mehlhorn AT Niemeyer P Kaschte K Muller L Finkenzeller G Hartl D Sudkamp NP Schmal H Differential effects of BMP-2 and TGF-beta1 on chondrogenic differentiation of adipose derived stem cells Cell Prolif 2007 40 809 823 10.1111/j.1365-2184.2007.00473.x 18021172 PMC6496220

[B8] Kim HD Valentini RF Retention and activity of BMP-2 in hyaluronic acid-based scaffolds in vitro J Biomed Mater Res 2002 59 573 584 10.1002/jbm.10011 11774316

[B9] Yamaoka H Asato H Ogasawara T Nishizawa S Takahashi T Nakatsuka T Koshima I Nakamura K Kawaguchi H Chung UI Cartilage tissue engineering using human auricular chondrocytes embedded in different hydrogel materials J Biomed Mater Res A 2006 78 1 11 16596585 10.1002/jbm.a.30655

[B10] Dell'Accio F De Bari C El Tawil NM Barone F Mitsiadis TA O'Dowd J Pitzalis C Activation of WNT and BMP signaling in adult human articular cartilage following mechanical injury Arthritis Res Ther 2006 8 R139 10.1186/ar2029 16893455 PMC1779445

[B11] Chubinskaya S Hurtig M Rueger DC OP-1/BMP-7 in cartilage repair Int Orthop 2007 31 773 781 10.1007/s00264-007-0423-9 17687553 PMC2266666

[B12] Brittberg M Winalski CS Evaluation of cartilage injuries and repair J Bone Joint Surg Am 2003 85-A Suppl 2 58 69 12721346 10.2106/00004623-200300002-00008

[B13] Steinwachs M New technique for cell-seeded collagen-matrix-supported autologous chondrocyte transplantation Arthroscopy 2009 25 208 211 10.1016/j.arthro.2008.10.009 19171282

[B14] Gillogly SD Myers TH Reinold MM Treatment of full-thickness chondral defects in the knee with autologous chondrocyte implantation J Orthop Sports Phys Ther 2006 36 751 764 17063837 10.2519/jospt.2006.2409

[B15] Brittberg M Lindahl A Nilsson A Ohlsson C Isaksson O Peterson L Treatment of deep cartilage defects in the knee with autologous chondrocyte transplantation N Engl J Med 1994 331 889 895 10.1056/NEJM199410063311401 8078550

[B16] Kreuz PC Erggelet C Steinwachs MR Krause SJ Lahm A Niemeyer P Ghanem N Uhl M Sudkamp N Is microfracture of chondral defects in the knee associated with different results in patients aged 40 years or younger? Arthroscopy 2006 22 1180 1186 10.1016/j.arthro.2006.06.020 17084294

[B17] Geborek P Saxne T Heinegard D Wollheim FA Measurement of synovial fluid volume using albumin dilution upon intraarticular saline injection J Rheumatol 1988 15 91 94 3280796

[B18] Chubinskaya S Frank BS Michalska M Kumar B Merrihew CA Thonar EJ Lenz ME Otten L Rueger DC Block JA Osteogenic protein 1 in synovial fluid from patients with rheumatoid arthritis or osteoarthritis: relationship with disease and levels of hyaluronan and antigenic keratan sulfate Arthritis Res Ther 2006 8 R73 10.1186/ar1947 16646979 PMC1526629

[B19] Honsawek S Chayanupatkul M Tanavalee A Sakdinakiattikoon M Deepaisarnsakul B Yuktanandana P Ngarmukos S Relationship of plasma and synovial fluid BMP-7 with disease severity in knee osteoarthritis patients: a pilot study Int Orthop 2009 33 1171 1175 10.1007/s00264-009-0751-z 19301001 PMC2898966

[B20] Lohmander LS Dahlberg L Ryd L Heinegard D Increased levels of proteoglycan fragments in knee joint fluid after injury Arthritis Rheum 1989 32 1434 1442 10.1002/anr.1780321113 2554931

[B21] Lysholm J Gillquist J Evaluation of knee ligament surgery results with special emphasis on use of a scoring scale Am J Sports Med 1982 10 150 154 10.1177/036354658201000306 6896798

[B22] Edwards DJ Brown JN Roberts SN Paterson RS Long-term results of anterior cruciate ligament reconstruction using ilio-tibial tract and semitendinosis tendon Knee 2000 7 87 93 10.1016/S0968-0160(00)00035-1 10788770

[B23] Noyes FR Barber SD Mooar LA A rationale for assessing sports activity levels and limitations in knee disorders Clin Orthop Relat Res 1989 238 249 2670388

[B24] Saris DB Vanlauwe J Victor J Haspl M Bohnsack M Fortems Y Vandekerckhove B Almqvist KF Claes T Handelberg F Characterized chondrocyte implantation results in better structural repair when treating symptomatic cartilage defects of the knee in a randomized controlled trial versus microfracture Am J Sports Med 2008 36 235 246 10.1177/0363546507311095 18202295

[B25] Glansbeek HL van Beuningen HM Vitters EL Morris EA van der Kraan PM van den Berg WB Bone morphogenetic protein 2 stimulates articular cartilage proteoglycan synthesis in vivo but does not counteract interleukin-1alpha effects on proteoglycan synthesis and content Arthritis Rheum 1997 40 1020 1028 10.1002/art.1780400605 9182911

[B26] Grunder T Gaissmaier C Fritz J Stoop R Hortschansky P Mollenhauer J Aicher WK Bone morphogenetic protein (BMP)-2 enhances the expression of type II collagen and aggrecan in chondrocytes embedded in alginate beads Osteoarthritis Cartilage 2004 12 559 567 10.1016/j.joca.2004.04.001 15219571

[B27] Kim DJ Moon SH Kim H Kwon UH Park MS Han KJ Hahn SB Lee HM Bone morphogenetic protein-2 facilitates expression of chondrogenic, not osteogenic, phenotype of human intervertebral disc cells Spine (Phila Pa 1976) 2003 28 2679 2684 14673369 10.1097/01.BRS.0000101445.46487.16

[B28] Li J Yoon ST Hutton WC Effect of bone morphogenetic protein-2 (BMP-2) on matrix production, other BMPs, and BMP receptors in rat intervertebral disc cells J Spinal Disord Tech 2004 17 423 428 10.1097/01.bsd.0000112084.85112.5d 15385883

[B29] Blaney Davidson EN Vitters EL van Lent PL van de Loo FA van den Berg WB van der Kraan PM Elevated extracellular matrix production and degradation upon bone morphogenetic protein-2 (BMP-2) stimulation point toward a role for BMP-2 in cartilage repair and remodeling Arthritis Res Ther 2007 9 R102 10.1186/ar2305 17922907 PMC2212581

[B30] Scharstuhl A Vitters EL van der Kraan PM van den Berg WB Reduction of osteophyte formation and synovial thickening by adenoviral overexpression of transforming growth factor beta/bone morphogenetic protein inhibitors during experimental osteoarthritis Arthritis Rheum 2003 48 3442 3451 10.1002/art.11328 14673995

[B31] Flechtenmacher J Huch K Thonar EJ Mollenhauer JA Davies SR Schmid TM Puhl W Sampath TK Aydelotte MB Kuettner KE Recombinant human osteogenic protein 1 is a potent stimulator of the synthesis of cartilage proteoglycans and collagens by human articular chondrocytes Arthritis Rheum 1996 39 1896 1904 10.1002/art.1780391117 8912513

[B32] Loeser RF Chubinskaya S Pacione C Im HJ Basic fibroblast growth factor inhibits the anabolic activity of insulin-like growth factor 1 and osteogenic protein 1 in adult human articular chondrocytes Arthritis Rheum 2005 52 3910 3917 10.1002/art.21472 16320338 PMC1482464

[B33] Nishida Y Knudson CB Eger W Kuettner KE Knudson W Osteogenic protein 1 stimulates cells-associated matrix assembly by normal human articular chondrocytes: up-regulation of hyaluronan synthase, CD44, and aggrecan Arthritis Rheum 2000 43 206 214 10.1002/1529-0131(200001)43:1<206::AID-ANR25>3.0.CO;2-1 10643717

[B34] Chubinskaya S Hakimiyan A Pacione C Yanke A Rappoport L Aigner T Rueger DC Loeser RF Synergistic effect of IGF-1 and OP-1 on matrix formation by normal and OA chondrocytes cultured in alginate beads Osteoarthritis Cartilage 2007 15 421 430 10.1016/j.joca.2006.10.004 17126570 PMC1894688

[B35] Loeser RF Pacione CA Chubinskaya S The combination of insulin-like growth factor 1 and osteogenic protein 1 promotes increased survival of and matrix synthesis by normal and osteoarthritic human articular chondrocytes Arthritis Rheum 2003 48 2188 2196 10.1002/art.11209 12905472

[B36] Chubinskaya S Kumar B Merrihew C Heretis K Rueger DC Kuettner KE Age-related changes in cartilage endogenous osteogenic protein-1 (OP-1) Biochim Biophys Acta 2002 1588 126 134 12385776 10.1016/s0925-4439(02)00158-8

[B37] Merrihew C Kumar B Heretis K Rueger DC Kuettner KE Chubinskaya S Alterations in endogenous osteogenic protein-1 with degeneration of human articular cartilage J Orthop Res 2003 21 899 907 10.1016/S0736-0266(03)00055-X 12919879

[B38] Chubinskaya S Merrihew C Cs-Szabo G Mollenhauer J McCartney J Rueger DC Kuettner KE Human articular chondrocytes express osteogenic protein-1 J Histochem Cytochem 2000 48 239 250 10639490 10.1177/002215540004800209

[B39] Haaijman A Burger EH Goei SW Nelles L ten Dijke P Huylebroeck D Bronckers AL Correlation between ALK-6 (BMPR-IB) distribution and responsiveness to osteogenic protein-1 (BMP-7) in embryonic mouse bone rudiments Growth Factors 2000 17 177 192 10.3109/08977190009001067 10705576

[B40] Hauburger A von Einem S Schwaerzer GK Buttstedt A Zebisch M Schraml M Hortschansky P Knaus P Schwarz E The pro-form of BMP-2 interferes with BMP-2 signalling by competing with BMP-2 for IA receptor binding Febs J 2009 276 6386 6398 10.1111/j.1742-4658.2009.07361.x 19804412

[B41] Felson DT The sources of pain in knee osteoarthritis Curr Opin Rheumatol 2005 17 624 628 10.1097/01.bor.0000172800.49120.97 16093843

[B42] Feng JQ Guo FJ Jiang BC Zhang Y Frenkel S Wang DW Tang W Xie Y Liu CJ Granulin epithelin precursor: a bone morphogenic protein 2-inducible growth factor that activates Erk1/2 signaling and JunB transcription factor in chondrogenesis Faseb J 24 1879 1892 10.1096/fj.09-144659 20124436 PMC2874481

[B43] Gouttenoire J Bougault C Aubert-Foucher E Perrier E Ronziere MC Sandell L Lundgren-Akerlund E Mallein-Gerin F BMP-2 and TGF-beta1 differentially control expression of type II procollagen and alpha 10 and alpha 11 integrins in mouse chondrocytes Eur J Cell Biol 89 307 314 10.1016/j.ejcb.2009.10.018 20129696 PMC3791590

[B44] Heinecke K Seher A Schmitz W Mueller TD Sebald W Nickel J Receptor oligomerization and beyond: a case study in bone morphogenetic proteins BMC Biol 2009 7 59 10.1186/1741-7007-7-59 19735544 PMC2749821

[B45] Steinert AF Proffen B Kunz M Hendrich C Ghivizzani SC Noth U Rethwilm A Eulert J Evans CH Hypertrophy is induced during the in vitro chondrogenic differentiation of human mesenchymal stem cells by bone morphogenetic protein-2 and bone morphogenetic protein-4 gene transfer Arthritis Res Ther 2009 11 R148 10.1186/ar2822 19799789 PMC2787261

